# Searching for the Best Machine Learning Algorithm for the Detection of Left Ventricular Hypertrophy from the ECG: A Review

**DOI:** 10.3390/bioengineering11050489

**Published:** 2024-05-15

**Authors:** Simon W Rabkin

**Affiliations:** Department of Medicine, Division of Cardiology, University of British Columbia, 9th Floor 2775 Laurel St., Vancouver, BC V5Z 1M9, Canada; simon.rabkin@ubc.ca; Tel.: +1-(604)-875-5847; Fax: +1-(604)-875-5849

**Keywords:** left ventricular hypertrophy, electrocardiogram, machine learning, artificial intelligence

## Abstract

**Background:** Left ventricular hypertrophy (LVH) is a powerful predictor of future cardiovascular events. **Objectives:** The objectives of this study were to conduct a systematic review of machine learning (ML) algorithms for the identification of LVH and compare them with respect to the classical features of test sensitivity, specificity, accuracy, ROC and the traditional ECG criteria for LVH. **Methods:** A search string was constructed with the operators “left ventricular hypertrophy, electrocardiogram” AND machine learning; then, Medline and PubMed were systematically searched. **Results**: There were 14 studies that examined the detection of LVH utilizing the ECG and utilized at least one ML approach. ML approaches encompassed support vector machines, logistic regression, Random Forest, GLMNet, Gradient Boosting Machine, XGBoost, AdaBoost, ensemble neural networks, convolutional neural networks, deep neural networks and a back-propagation neural network. Sensitivity ranged from 0.29 to 0.966 and specificity ranged from 0.53 to 0.99. A comparison with the classical ECG criteria for LVH was performed in nine studies. ML algorithms were universally more sensitive than the Cornell voltage, Cornell product, Sokolow-Lyons or Romhilt-Estes criteria. However, none of the ML algorithms had meaningfully better specificity, and four were worse. Many of the ML algorithms included a large number of clinical (age, sex, height, weight), laboratory and detailed ECG waveform data (P, QRS and T wave), making them difficult to utilize in a clinical screening situation. **Conclusions**: There are over a dozen different ML algorithms for the detection of LVH on a 12-lead ECG that use various ECG signal analyses and/or the inclusion of clinical and laboratory variables. Most improved in terms of sensitivity, but most also failed to outperform specificity compared to the classic ECG criteria. ML algorithms should be compared or tested on the same (standard) database.

## 1. Introduction

A number of different groups have proposed machine learning models to evaluate ECG with or without additional clinical and laboratory data to construct an approach to identify left ventricular hypertrophy (LVH). LVH, or an increased left ventricular mass, is a powerful predictor of future cardiovascular events [1,2,3]. LVH can serve as a marker for the severity of (occult) cardiovascular disease, thereby identifying an increased risk of stroke or, more directly, by limiting myocardial perfusion, leading to myocardial ischemia and serious cardiac arrhythmias [4,5,6,7,8]. The ECG has been used for decades as an indicator of the presence of LVH, with increased QRS voltage being considered to be a marker for increased left ventricular mass [9,10,11,12,13,14,15,16]. Although the ECG QRS voltage criteria are not a highly sensitive indicator of LVH [17,18,19,20,21,22,23,24,25], the importance of predicting the presence of LVH and the imperative of cost efficiency, i.e., utilization of a low-cost ECG compared to a more expensive echocardiogram or MRI, has focused attention on how to extract more precise indicators of LVH from ECGs. This imperative is underscored by the use of a 12-lead ECG as part of the basic assessment of patients with cardiovascular disease [26,27].

Because of the importance of LVH identification, the ML disparate approaches and the variables considered in each approach, a review of this field has become increasingly needed.

A meta-analysis, to construct a single estimate or effect size, is not realistic in fields such as machine learning, when different input variables and analytic techniques are employed by utilizing different algorithms on different datasets. Hence, an in-depth review is the best approach to evaluate different ML algorithms. The objectives of this study were to conduct a review of machine learning algorithms for the identification of LVH with respect to the classical features of sensitivity, specificity and accuracy. It also aimed to assess how each ML algorithm compares to the traditional ECG criteria for LVH, specifically Cornell voltage [13], Cornell product [28], Sokolow-Lyons [9] and Romhilt-Estes [29] criteria.

## 2. Methods

### 2.1. Literature Search

A search string was constructed using terms connected with Boolean operators “left ventricular hypertrophy AND electrocardiogram or ECG” AND machine learning to identify articles reporting a machine learning approach for the diagnosis of LVH. Medline and PubMed were systematically searched from their date of inception through to 31 October 2023. Preferred Reporting Items for Reviews and Meta-Analysis (PRISMA) was used to conduct the search [30] (Supplementary Figure S1).

Article titles and abstracts were assessed for full-text review. Papers on hypertrophic obstructive cardiomyopathy were excluded because this entity represents an asymmetric cardiac hypertrophy, which would alter ECG voltage in a different manner. The exclusion criteria were as follows: non-English studies, non-primary studies, studies without full texts, studies that have insufficient data for analysis, non-human studies and studies unrelated to the investigated topic. The review was not registered, and protocol is not available for access.

### 2.2. Data Extraction and Classification

Data extraction was performed by one reviewer. The following items were collected from each article: author, year of publication, recruitment center or clinical trial sampled, sample size, age and sex of participants, definition of LVH and its method of assessment. Reported sensitivity, specificity, positive predictive value, negative predictive value, area under the receiver operating curve (ROC), overall accuracy and F1 score were extracted. Input variables and the ML techniques utilized were also extracted.

## 3. Results

There were 14 studies that examined the detection of left ventricular hypertrophy with an approach utilizing the ECG and utilized at least one machine language approach (Table 1). Some details of the study population characteristics and input variables are summarized in Table 1.

Lin and Liu evaluated data from 2196 men, aged 17 to 45 years of age, who were in the military, and used the support vector machine (SVM) classifier as the machine learning method [31]. The prevalence of echocardiographic LVH was about 6.5%. Thirty-one input variables were utilized that included three clinical ones, age, body height and body weight, and 28 ECG parameters, such as heart rate, the durations of P wave, PR interval, QRS interval, QT interval and QTc interval in Lead II and the axes of the P, QRS and T waves in Lead II, and the voltages of R waves in all Limb Leads I, II, III, aVR, aVL, aVF and S wave in Lead aVL, and the voltages of R and S waves in all precordial leads V1–V6 [31]. The model had high sensitivity (86.7%). 

Sparapani et al. evaluated 3774 participants from MESA (Multi-Ethnic Study of Atherosclerosis), free of clinically apparent cardiovascular disease at enrollment, using ECG and participant characteristics to predict LV mass from cardiac magnetic resonance imaging [32]. There were four global ECG measurements (PR interval, P axis, QRS interval and QRS axis) plus 552 amplitude and duration measurements per ECG, which resulted in 556 ECG variables. The machine learning technique Bayesian Additive Regression Trees (BART) was used [32]. This model showed the highest sensitivity (29.0%), greater than the other criteria, including the Sokolow-Lyon criterion (21.7%), Peguero-Lo Presti (14.5%), Cornell voltage product (10.1%) and Cornell voltage (5.8%). The specificity was >93% for all criteria [32].

Garza-Salazar et al. conducted an observational, retrospective case–control study that included data from a representative sample of consecutive adult patients who underwent an ECG and an echocardiogram at their institution [33]. They evaluated 432 patients, of whom 47% had LVH [33]. The ECG variables included S-wave voltage and R-wave voltage in all ECG leads (I, II, III, aVL, aVF, aVR and V1-V6), P-wave duration and voltage in the V1 lead, left atrial enlargement, QRS complex duration in lead V1, QRS axis (using leads I and aVL), intrinsicoid deflection in lead V6 and “ST strain” (downward ST depression and asymmetric T-wave inversion) [33]. The logistic regression (LR) model was used as well as a supervised ML algorithm to create a multilevel binary decision tree, using the ECG *features* that provided the greatest information to classify patients as having LVH [33]. Their five-level binary decision tree used only six predictive variables and had an accuracy of 71.4%, a sensitivity of 79.6% and specificity of 53% [33].

De la Garza Salazar et al. reported another observational, retrospective, case–control study on 439 patients who underwent an echocardiogram and an ECG [35]. Sixteen ECG parameters, including T voltage in lead I, peak-to-peak QRS distance in aVL (>1.235 mV) and peak-to-peak QRS distance in aVF (>0.178 mV), were fed into a C5.0 ML algorithm, a method that defines a decision tree structure model (or criteria). Their model had an accuracy of 70.5%, a sensitivity of 74.3% and a specificity of 68.7%.

Kwon et al. conducted a retrospective cohort study of 12,648 patients who underwent 12-lead ECG and echocardiography [34]. LVH was present in 21% of the group. An ensemble neural network (ENN) combining a convolutional (CNN) and deep neural network (DNN) was developed. Two other machine learning-based algorithms—LR and RF—were also developed. The model was developed using 3162 ECGs from 3162 patients. They used four clinical variables (age, sex, weight and height) and ECG features, such as heart rate, presence of atrial fibrillation or flutter, QT interval, QTc, QRS duration, R-wave axis and T-wave axis. In addition, they used raw ECG data with 5000 numbers from each of the 12 leads. The area under the ROC curve for ENN was 0.880, which significantly outperformed the Romhilt-Estes point system, Cornell voltage criteria and the Sokolow-Lyon criteria [34].

Lim et al. examined the ECGs and echocardiograms of 17,310 male military conscripts, aged 16 to 23 years [39]. The prevalence of echocardiographic LVH was 0.82%. Several machine learning models (Logistic Regression, GLMNet, Random Forests and Gradient Boosting Machines) were used. Their clinical variables were body weight, height, body fat percentage and systolic blood pressure. Their ECG variables included QT interval, mean QRS duration and R wave in lead I, ECG parameters not used in the classical criteria but deemed important to the machine learning algorithms, both when ECG parameters alone were included and when all predictive parameters were included. Considering AUC, ML methods achieved superior performance: logistic regression (0.811), GLMNet (0.873), Random Forest (0.824) and Gradient Boosting Machines (0.800).

Two studies used the UK Biobank with individuals aged 40 to 69 years, with a mean age of 64 years, of which 52% were female, who had LV mass index assessed by MRI [36]. Khurshid et al. also tested a Massachusetts General/Brigham Hospital database, but more information was available for the UK Biobank so it was selected in this analysis. Khurshid et al. trained an ML model on 32, 239 participants [36]. The input variables were demographic factor, age, sex, race, height, weight and body mass index (BMI), plus ECG waveform data. Their model had a sensitivity of 34% and a specificity of 96% [36].

Naderi et al. also explored the UK Biobank [43]. There was a low prevalence of LVH, specifically 1.5%. Demographic factors included age, sex and race, and physical measurements included height, weight and body mass index (BMI). Clinical variables included blood pressure, and 23 ECG variables used the independent ECG leads (I, II, V1–6). ECG variables consisted of ECG waveform data. Three supervised machine learning algorithms, logistic regression (LR), support vector machine (SVM) and Random Forest (RF), were used. For the SVM classifier, the Gaussian kernel function was applied to deal with potential non-linear data. The three models were comparable in classifying LVH. Classification of LVH with logistic regression had an accuracy of 81%, sensitivity of 70%, specificity of 81% and an AUC of 0.86. Analysis with SVM showed 81% accuracy, sensitivity of 72%, specificity of 81% and AUC of 0.85. RF analysis showed 72% accuracy, sensitivity of 74%, specificity of 72% and AUC of 0.83 [43].

Sabovčik et al. evaluated 1407 individuals (mean age 51 years, 51% women), randomly recruited from the general population, of whom an echocardiographically determined LV mass was present in 19% [37]. A large number of clinical and laboratory variables (blood count, blood glucose, lipids, renin activity, leptin, insulin, aldosterone and cortisol) were used. From the ECG tracing, the onsets, amplitudes and intervals of P waves, QRS complexes and T waves were extracted. They used five standard ML methods, XGBoost, AdaBoost, RF, SVM and LR, to build classifiers based on 67 clinical, biochemical and ECG variables. A high area under the ROC was found for XGBoost (0.785), RF classifiers (0.783), AdaBoost (0.771), SVM (0.783) and LR (0.783) for predicting LVH. Age, body mass index, different components of blood pressure, history of hypertension, antihypertensive treatment and various electrocardiographic variables were the top features for predicting LVH [37].

Angelaki et al. evaluated 528 patients with and without essential hypertension but no other indications of cardiovascular disease [38]. LVH, assessed by echocardiogram, was present in 16.8% of cases. Clinical variables were used. ECG waveform measurements from each lead included peak voltages, area of the QRS complex, planar frontal QRS-T angle and QTc duration. A Random Forest ML algorithm consisting of a collection of de-correlated decision trees was used. They calculated SHAP (SHapley Additive exPlanations). Hypertension, age and BMI were the most significant factors predicting the presence of LVH. The area under the QRS complex summed over all 12 leads, the Planar Frontal QRS-T angle and QTc duration, among others, was important in predicting risk. For the identification of LVH, their model noted 87% accuracy, 75% specificity, 97% sensitivity and area under the receiver operating curve (AUC/ROC) of 0.91 [38]. However, some of the patients did not have LVH but rather concentric remodeling [38].

Kokubo et al. analyzed data from patients aged 18 years or older, with a mean age 64.2 years, 57% men, who had an echocardiogram and ECG at The University of Tokyo Hospital [42]. LVH was defined as an LVMI > 101 g/m^2^ for men and > 85 g/m^2^ for women, consistent with recommendations for the Japanese population, and was present in 16.5% of cases [42]. The data were derived from a training set of 12,008 persons. Nineteen factors—clinical (age, sex, height and weight) and ECG features (heart rate, rhythm, pr interval, QT interval, QRS axis, P wave axis as well as QRS voltages in leads V1, V2, V5 and V6)—were used as input variables. They developed an ensemble neural network (ENN) model, which consisted of a convolutional neural network (CNN) and a deep neural network (DNN) as well as a LR and RF approaches to detect LVH. For the detection of LVH, the area under the ROC curve was 0.784 for the deep learning model, which was significantly greater than that of the LR, RF or conventional ECG criteria [42].

Zhao et al. utilized data from 3120 patients who had an echocardiogram and an ECG within one week after hospital admission [40]. The input variables included clinical factors, such as age, sex and medical history; laboratory factors, such as hemoglobin, platelet count, lipids, creatinine, Na, K^+^; ECG factors, such as R in AVL, V5 and V6, and S in V1 and V3. The ECG final dataset included 36,350 ECG segments in the control and LVH groups. They constructed and built a deep learning (DL) model based on convolutional neural network–long short-term memory (CNN-LSTM) to detect LVH. LVH was predicted by the CNN-LSTM model with an area under the curve (AUC) of 0.62, with a sensitivity of 68% and specificity of 57%. The CNN-LSTM model predicted LVH by 12-lead ECG performed better in male than female patients [40].

Sammani et al. developed an ML algorithm for echocardiographically detected LVH that utilized a variety of clinical factors (age, systolic blood pressure and body surface area) and over 20 ECG data variables (P, QRS and T wave axes, PR, QRS, QT and QTc durations, peak amplitudes of P, Q, R, S and T waves in three different ECG leads) [41]. There were 26,954 subjects (median age 61 years, 55% male), of whom 0.8% had LVH, and of those with LVH, a very small number had amyloidosis; only two had Anderson-Fabry Disease. XGBoost was the only machine learning logarithm used [41].

Liu et al. studied 952 individuals, mainly men, from a military hospital and used a back-propagation neural network (BPN) on 24 features, which consisted of R peak and S valley amplitudes, automatically obtained from the output of the ECG signal. This group found a prevalence of 18% with echocardiographic LVH. Their combination of sensitivity and specificity was the highest of any approach [44].

**Figure 1:**
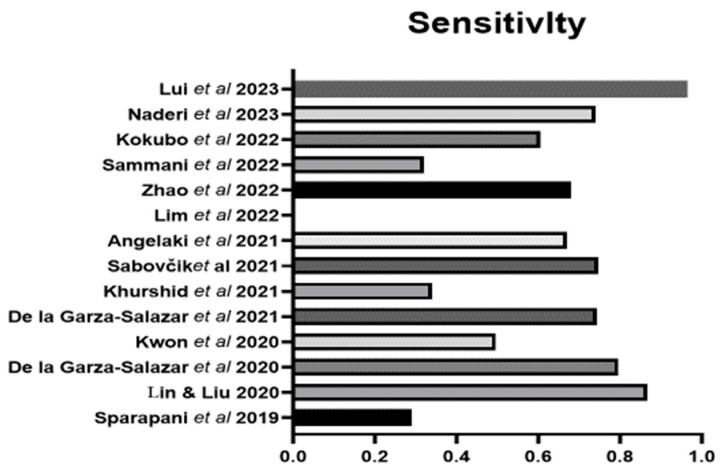
The sensitivity of the ML algorithms for LVH. Lin & Lui 2020 [31], Sparapani et al., 2019 [32], De la Gar-za-Salazar et al., 2020 [33], Kwon et al., 2020 [34], De la Gar-za-Salazar et al., 2021 [35], Khurshid et al., 2021 [36], Sabovčik et al., 2021 [37], Angelaki et al., 2021 [38], Lim et al., 2021 [39], Zhao et al., 2022 [40], Sammani et al., 2022 [41], Kokubo et al., 2022 [42], Naderi et al., 2023 [43], Liu et al., 2023 [44].

**Figure 2:**
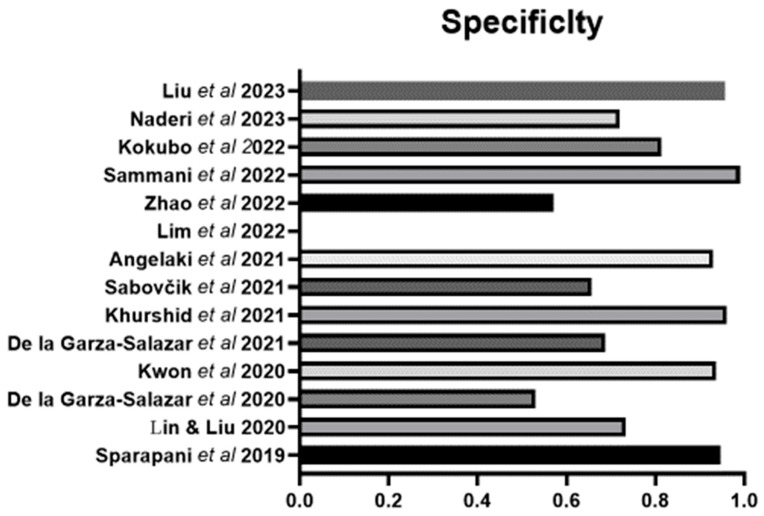
The specificity of the ML algorithms for LVH. Lin & Lui 2020 [31], Sparapani et al., 2019 [32], De la Gar-za-Salazar et al., 2020 [33], Kwon et al., 2020 [34], De la Gar-za-Salazar et al., 2021 [35], Khurshid et al., 2021 [36], Sabovčik et al., 2021 [37], Angelaki et al., 2021 [38], Lim et al., 2021 [39], Zhao et al., 2022 [40], Sammani et al., 2022 [41], Kokubo et al., 2022 [42], Naderi et al., 2023 [43], Liu et al., 2023 [44].

Sensitivity and specificity were reported in 13 of the 14 studies. There was a wide range of sensitivity for the ML approaches across all studies. The range is from 0.29 to 0.966 (Figure 1). The highest sensitivity was 0.966 using the algorithm proposed by Liu et al., 2023 [44], followed by 0.867 using the algorithm proposed by Lin and Liu [31], followed by the one proposed by De la Garza-Salazar et al. [33]. Specificity ranged from 0.53 to 0.99 (Figure 2). The highest specificity was found using the algorithm proposed by Sammani et al. [41], followed closely by that of Liu et al. [44] and then Khurshid et al. [36].

**Figure 3:**
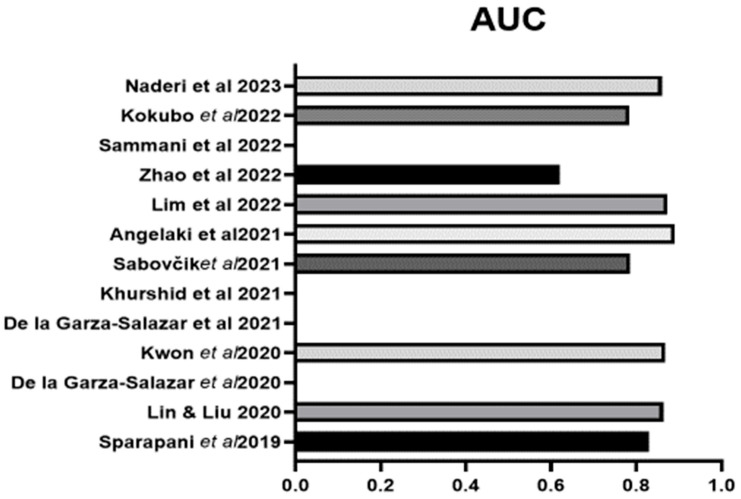
The AUC of the ML algorithms for LVH in those studies that reported such data. Lin & Lui 2020 [31], Sparapani et al., 2019 [32], De la Gar-za-Salazar et al., 2020 [33], Kwon et al., 2020 [34], De la Gar-za-Salazar et al., 2021 [35], Khurshid et al., 2021 [36], Sabovčik et al., 2021 [37], Angelaki et al., 2021 [38], Lim et al., 2021 [39], Zhao et al., 2022 [40], Sammani et al., 2022 [41], Kokubo et al., 2022 [42], Naderi et al., 2023 [43].

**Figure 4:**
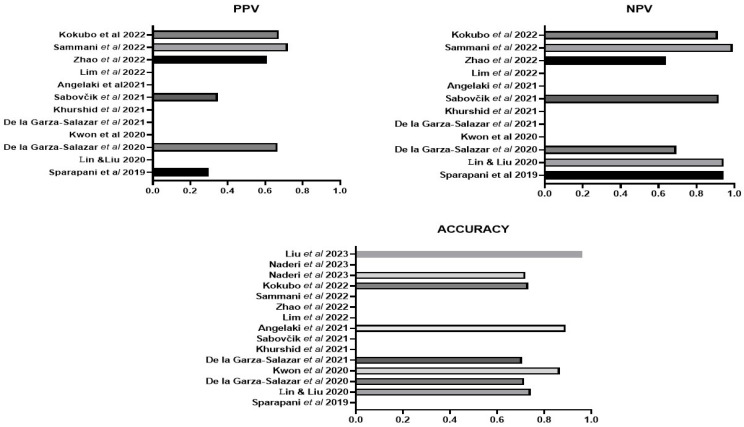
The positive predictive value (PPV), negative predictive value (NPV) and accuracy of the ML algorithms for LVH in those studies that reported such data. Lin & Lui 2020 [31], Sparapani et al., 2019 [32], De la Gar-za-Salazar et al., 2020 [33], Kwon et al., 2020 [34], De la Gar-za-Salazar et al., 2021 [35], Khurshid et al., 2021 [36], Sabovčik et al., 2021 [37], Angelaki et al., 2021 [38], Lim et al., 2021 [39], Zhao et al., 2022 [40], Sammani et al., 2022 [41], Kokubo et al., 2022 [42], Naderi et al., 2023 [43], Liu et al., 2023 [44].

An overall assessment indicated by AUC was reported in nine studies and ranged from 0.705 to 0.89, with the highest AUC reported for the algorithms of Angelaki et al. [38] followed by Kwon et al. [34] (Figure 3). Overall accuracy was detailed in eight studies, with the highest value of 0.961 from Liu et al. [44] followed by that of Angelaki et al. [38] (Figure 4). The next best was that of Kwon et al. [34]. Positive and negative predictive values were reported in less than one half, or only six studies (Figure 4). Four studies presented their F1 score, which is a composite indicator of sensitivity, the true-positive rate, taking into account false positives and false negatives. The values ranged from 0.294 [32] to 0.3314 [31] and 0458–0.488 (depending on the ML method) [37] to 0.64, which was the highest value and was reported by Zhao et al., 2022 [40].

Three studies used different ML algorithms and compared them. Kwon et al. found that their AI algorithm based on ENN significantly outperformed the DNN, CNN, RF and LR ones using AUC as the metric [34]. Using the same metric (AUC), Sabovcik et al. reported that XGBoost and RF classifiers exhibited a high area under the receiver operating characteristic curve, with values between 77.7% and 78.5%, for predicting LVH, and these approaches were better than AdaBoost, support vector machines and logistic regression [37]. They did not use an ENN approach. Kokubo et al. found values of 78.4% for the deep learning model (ENN), which was significantly higher than that of the logistic regression and Random Forest methods [42]. Thus, based on the two studies that used ENN, ENN offers a competitive advantage over other ML approaches [34,42].

Nine studies compared their ML approach to the classic ECG approach. The ML algorithm of Zhao et al. outperformed Cornell voltage criteria (AUC 0.57, sensitivity 48%, specificity 72%) and Sokolow-Lyon voltage (AUC 0.51, sensitivity 14%, specificity 96%). [40]. The ML algorithm proposed by Liu et al. reported sensitivity, specificity and accuracy values that were better than the Cornell voltage criteria, Sokolow-Lyons, Peguero, Framingham and Gubner criteria [44]. Of the two ML algorithms presented by De la Garza-Salazar et al., the first had better results than the Romhilt-Estes score, with an accuracy of 61.3%, a sensitivity of 23.2% and a specificity of 94.8% [33], while the second one had an accuracy better than Romhilt-Estes (57%), Cornell (59%) and Sokolow-Lyon (53.9%) [35].

**Figure 5:**
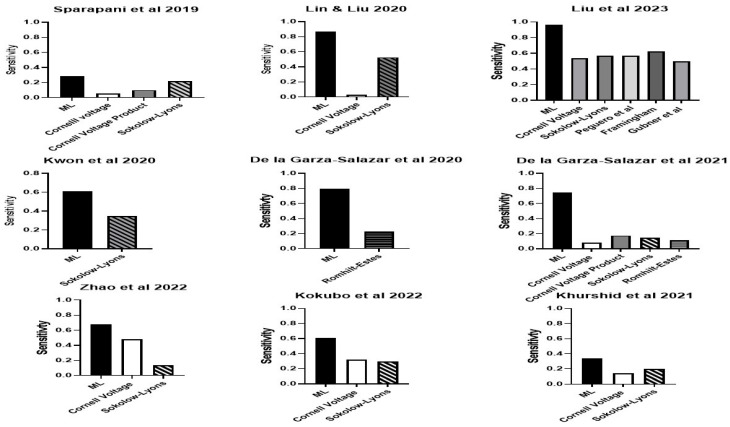
The sensitivity of the ML algorithms for LVH compared to standard ECG LVH criteria in studies that reported such data. Lin & Lui 2020 [31], Sparapani et al., 2019 [32], De la Gar-za-Salazar et al., 2020 [33], Kwon et al., 2020 [34], De la Gar-za-Salazar et al., 2021 [35], Khurshid et al., 2021 [36], Zhao et al., 2022 [40], Kokubo et al., 2022 [42], Liu et al., 2023 [44].

**Figure 6:**
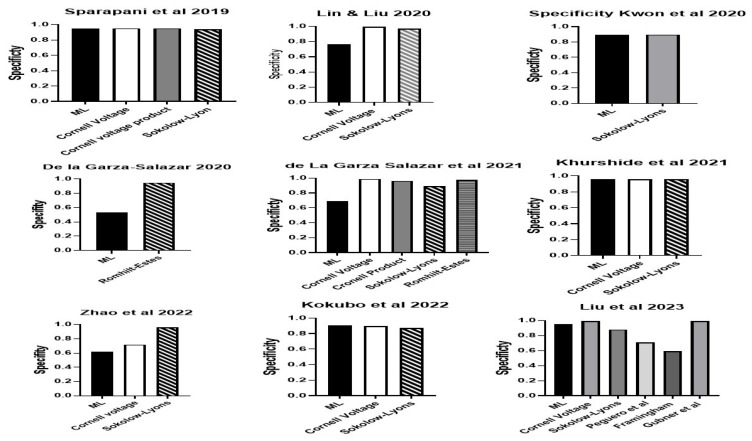
The specificity of the ML algorithms for LVH compared to standard ECG LVH criteria in studies that reported such data. Lin & Lui 2020 [31], Sparapani et al., 2019 [32], De la Gar-za-Salazar et al., 2020 [33], De la Gar-za-Salazar et al., 2021 [35], Khurshid et al., 2021 [36], Zhao et al., 2022 [40], Kokubo et al., 2022 [42], Liu et al., 2023 [44].

**Figure 7:**
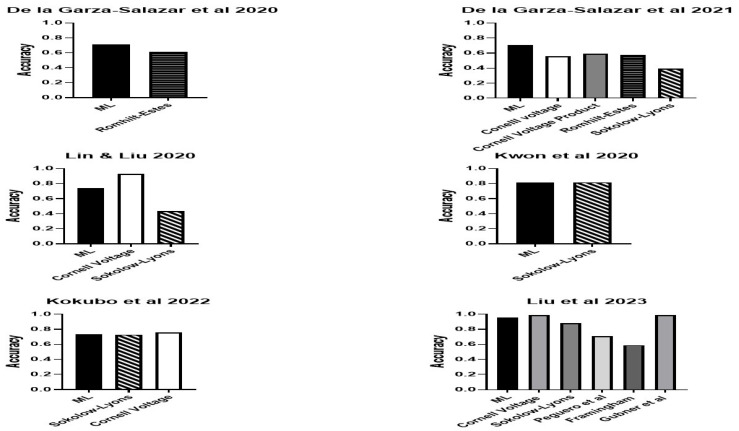
The accuracy of the ML algorithms for LVH compared to standard ECG LVH criteria in studies that reported such data. Lin & Lui 2020 [31], De la Gar-za-Salazar et al., 2020 [33], Kwon et al., 2020 [34], De la Gar-za-Salazar et al., 2021 [35], Kokubo et al., 2022 [42], Liu et al., 2023 [44].

Eight studies reported better sensitivity for their ML algorithm compared to assessment with the Romhilt-Estes point system, Cornell voltage criteria or Sokolow-Lyon criteria (Figure 5). Four of the eight studies reported a specificity of equal to or better than the classic ECG criteria [32,34,36,42] (Figure 6). For some ML algorithms, specificity was higher than the classic ECG criteria, while others did not find a significant difference. Seven studies reported better AUC for their ML algorithm compared to an assessment with the Romhilt-Estes point system, Cornell voltage criteria or Sokolow-Lyon criteria (Figure 7).

Several studies listed the important factors in their ML models. Ignoring the QRS voltage, Lin and Liu reported that there were other significant predictors of LVH, including age, heart rate, PR interval, uncorrected QT interval and QRS axis in Lead II [31]. Systolic and diastolic BP values were in the top-40 predictors of LVH in the algorithm proposed by Naderi et al. [43]. Age and blood pressure were key predictors of LVH in the ML model of Sammani et al., along with P- and T-wave characteristics [41]. Age, waist circumference, different components of BP, history of hypertension, serum renin and antihypertensive treatment were the top predictors of LVH in the algorithm of Sabovcik et al. [37].

## 4. Discussion

This study demonstrates the wide variety of machine learning techniques that have been used to assess the presence of an increased left ventricular mass or cardiac hypertrophy. It demonstrates the differences in sensitivity, specificity and predictive accuracy between ML algorithms. It further identifies large differences in the input variables between algorithms. These differences underscore the necessity to conduct an in-depth evaluation.

The sources of datasets for left ventricular hypertrophy from the ECG in the literature varied widely between studies. Two studies derived data from military recruits, who were essentially young men with a low prevalence of LVH [31,39]. There were three population-based studies, with two studies on the same UK database, with a greater prevalence of LVH [32,36,37,43]. There were eight hospital-based studies, which had, on average, the oldest mean age and the highest prevalence of LVH, with the proportion of men ranging from 42 to 64% [33,34,35,38,40,41,42] and one military hospital with a predominance of men (90%) [44]. Overall, the proportion of men and women varied greatly but mainly because of the predominance of men in the studies of young military recruits and in a military hospital. Young male military recruits may not be generalizable to the general population or to older patients admitted to hospital. The prevalence of LVH between studies ranged from 0.8 to 48% and may have influenced the precision of LVH detection. The majority of studies used echocardiograms to assess the prevalence of LVH, but the LVH criteria varied between studies in Asian or European populations.

ML algorithms may be differentiated by the manner in which they select the boundary that distinguishes different groupings. SVM was used by several groups [31,37,43]. The SVM classifier can use linear or non-linear functions, although linear functions are usually selected. The decision boundary in this method is called the maximum margin classifier, maximum margin hyperplane or the maximum margin hyper plane [45]. Other studies relied on logistic regression [33], a simpler method that tries to maximize the conditional likelihoods; however, it is more prone to outliers than SVMs, which mostly prioritize the points that are closest to the decision boundary. However, LR and SVM often yield similar results [46]. Some studies used RF [37,39,43]. Random forests are a classification algorithm using an ensemble of decision trees, such that each tree depends on the values of a random vector sampled independently, and the generalizability depends on the strength of each tree and the correlation between them [47]. In several clinical diagnosis conditions, RF showed the highest accuracy followed by SVM [48].

Angelaki et al. used SHAP (SHapley Additive exPlanations), a game theoretic approach that connects optimal credit allocation with local explanations, using the classic Shapley values from game theory and their related extensions [38]. A number of studies used multiple ML algorithms [37,39]. Some investigators employed deep learning methods [34,40,42]. The explosive growth of deep learning for ECG data led to the conclusion that a hybrid architecture of a convolutional neural network and recurrent neural network ensemble yielded the best results [49]. However, there are some new challenges and problems related to interpretability, scalability, and efficiency, in addition to differences in the perspectives of datasets and methods [49]. This hybrid combination has been used in a few studies for LVH detection [34,42].

Liu et al. reported both very high sensitivity and specificity. Usually, the higher a test sensitivity, the lower its specificity. They used detailed QRS analysis, but other studies that did not attain as a high a sensitivity and specificity also used detailed ECG signal analysis [43]; for example, Zhao et al. had 36,350 ECG segments in their final dataset [40], and another ML algorithm used 552 amplitude and duration measurements per ECG [32]. The findings of Liu et al. [44] showed both very high sensitivity and specificity, but this may relate to their decision that they had too few LVH cases “for designing a machine-learning model. Therefore, the beat segmentation method, Pan-Tompkins technique was performed to increase the ECG data amount to improve the detection performances” [44].

The crucial test of the ML algorithms is the comparative ability to predict LVH. The best or highest sensitivity was the algorithm proposed by Liu et al. [44], followed by Lin and Liu [31] and then by De la Garza-Salazar et al. [33]. If one wants a specific diagnosis, the highest specificity was found using the algorithm proposed by Sammani et al. [41], followed closely by that of Liu et al. [44] and then Khurshid et al. [36]. However, algorithms with high specificity often have low sensitivity. Combining sensitivity and specificity using ROC curves suggests the best approach would be the algorithms of Angelaki et al. [38] followed by Kwon et al. [34]. Several studies compared different ML models to predict LVH [34,39,42]. The differences were usually not large. Two studies compared at least four ML approaches, and both found that ENN had the highest AUC; ENN offered a competitive advantage over other ML approaches [34,42]. Kokopo et al. developed an ensemble neural network (ENN) model, which consisted of a convolutional neural network (CNN) and a deep neural network (DNN) [42]. Kwan et al. used a deep neural network (DNN). Based on the two studies that used ENN, this ML approach (ENN) offered a competitive advantage over other ML approaches [34,42].

Comparisons with classical ECG criteria for LVH showed that ML algorithms were usually more sensitive than the standard Cornell voltage [13], Cornell product [28], Sokolow-Lyons [9] or Romhilt-Estes criteria [29] for the detection of LVH. In contrast, generally, the ML algorithms were not more specific than the classic criteria, as four ML algorithms were no better and four were worse than these classic criteria for LVH. The ML algorithms of Sparapani et al. [32], Kokubo et al. [42], Kwan et al. [34] and Khurshid et al. [36] had a specificity equal to the classical ECG criteria.

A major theoretical issue with the ML algorithms for the detection of LVH is the use of different kinds of input data. There are several lines of reasoning for the use of ML for LVH diagnosis. The first is whether ML can improve LVH detection based on QRS complexes and especially QRS voltage, which was historically the first attempt to electrocardiographically identify LVH [9]. The second approach is to utilize all aspects of the ECG signal. This was embodied by the work of Romhilt and Estes [29], who added QRS axis and ST-T waves to QRS voltage to identify LVH. As such, ML algorithms can point to the classical approach to justify the inclusion of other ECG factors. The incorporation of clinical factors becomes more problematic in ECG assessment. When age and history of hypertension are included, sensitivity increases markedly, but is that a fair test of the use of ECGs in diagnosis? The addition of an extensive list of clinical and laboratory variables further removes the question from the utility of the ECG but satisfies the question of how to more accurately predict the presence of LVH. For example, Sabovčik et al. inputted a large number of clinical and laboratory variables, including blood count, blood glucose, lipids, renin activity, leptin, insulin, aldosterone and cortisol [37]. Zhao et al. included the input variables of clinical factors, age, sex and medical history, as well as laboratory factors, like hemoglobin, PLT, lipids, creatinine sodium and potassium [40]. The inclusion of such extensive clinical and laboratory data precludes the use of the ECG as a screening test for the presence of LVH, as all the clinical and other laboratory data, which are usually not available, would have to be inputted to utilize the algorithms.

Studies on machine learning-based prediction models have been criticized because of poor methodological quality and a high risk of bias [50]. The criticism relates to the frequent failure ‘to report key information to help readers judge the methods and have a complete, transparent and clear picture of the …content of the model’ [51]. This criticism has some validity in the assessment of ML algorithms for the detection/diagnosis of LVH. These kinds of models, because of their complexity, have been labelled as a ‘black box’, certainly compared to regression-based models that can be more recognizable [51]. For example, it is challenging to compare algorithms that state they are derived from 24 features, which consist of R peak and S valley amplitudes automatically obtained from the output of an ECG signal [44] versus raw ECG data, with 5000 numbers from each of the 12 leads [34]. Recognizing the limitations of each of the studies, it is worth discussing the implications of the results. First, ML algorithms can improve the sensitivity of the ECG for the detection of LVH. Improving sensitivity is important for a screening technique, and the ECG fulfills that requirement. Second, simplicity warrants using an algorithm that only relies on ECG variables to add to the ECG interpretation with respect to LVH. Third, algorithms that were developed utilizing a neural network approach appear to offer a competitive advantage over other ML approaches.

There are several limitations of this analysis that warrant discussion. First, the studies usually utilized ML approaches from available ‘packages’. Wallace et al. cautioned that the “near-ubiquitous reliance on ‘out of bag’ approaches may provide ‘misleading results” [52]. Second, many of the algorithms use ECG variables from most or all of the ECG leads, but in LVH detection, the QRS criteria from multiple leads often provide similar data [53]. Third, not all publications provided the same outputs to compare accuracy, F1 or ROC data. Fourth, it is difficult to compare and select the ‘best’ ML algorithms when one algorithm employs an extensive list of laboratory variables and another uses only ECG factors. One is left with the question whether one approach would be better if it also included an extensive list of laboratory tests.

In summary, it is important to re-emphasize the potential of the ECG to identify LVH because LVH is a significant predictor of cardiovascular events [3,8,19,24] and because a better approach for LVH detection would be an important contribution. Several ML algorithms improve the sensitivity, but most do not improve specificity for LVH diagnosis compared to classical ECG criteria. Future research is needed to obtain a more standardized approach for the evaluation and comparison of all ML algorithms using the same dataset to determine the competitive advantage of each and identify the best one. In addition, the separation of LVH diagnosis into two stages—an ECG interpretation that uses an ML algorithm and a second step with a simple application—can add further clinical variables.

## Figures and Tables

**Table 1:** Summary of the studies, input variables and machine learning approaches.

Authors	Population	Country	Sample Size	Sex (%M)	Age (yrs)	Method LVH	Definition LVH	LVH	Variables	Machine Learning
Lin & Lui 2020 [31]	Military	Tawain	2196	100	26	Echocardiogram	≥116 g/m^2^	6.5%	31 parameters 3 clinical -age, body height, body weight	Support vector machine classifier (SVM)
									28 ECG parameters: duration P, PR, QRS, QT, QTc, P axis QRS axis, T axis plus	
									R amplitude in all 12 leads, S amplitude in avL, V1-6	
Sparapani et al., 2019 [32]	Multi-ethnic	USA	4714	46		MRI	95th percentile	NA	556 ECG variables: PR interval, P axis, QRS interval, QRS axis plus 552 amplitudes and durations per ECG	Bayesian additive regression tree
De la Garza-Salazar et al., 2020 [33]	Hospital	Mexico	432	56	67	Echocardiogram	>115 g/m^2^ (men)	48%	ECG p wave, QRS complex and ST waves	C5.0 supervised ML algorithm to create a multilevel binary decision tree,
							>95 g/m^2^ (women).			
Kwon et al., 2020 [34]	Hospital based	Korea	21,286	49	59	Echocardiogram	>132 g/m^2^ in men	21%	age, sex, weight, height and ECG features, heart rate, presence of atrial fibrillation or flutter, QT, QRS duration, R-wave axis, T-wave	ENN, LR and RF
							>109 g/m^2^ in women		‘Raw’ ECG data with 5000 numbers from each of the 12 leads.	
De la Garza-Salazar et al., 2021 [35]	Hospital	Mexico	439	NA	67	Echocardiogram	Presumed same as 2020	46%	ECG variables including T wave voltage in the lead I, peak-to-peak QRS distance (QRS PPK) in aVF, and peak-to-peak QRS distance in aVL	C5.0 supervised ML algorithm to create a multilevel binary decision tree,
Khurshid et al., 2021 [36]	UK data base	UK	32,239	47	64	MRI		2.6%		
Sabovčik et al., 2021 [37]	General population	Belgium	1407	49	51	Echocardiogram	>115 g/m^2^ (men)	19%	67 variables including clinical, ECG onsets, amplitudes and intervals of P waves, QRS-complexes, and T wave as well as	LR, XGBoost, Random Forest, AdaBoost, Support Vector Machines
							or 95 g/m^2^(women).		blood count, blood glucose, lipid profile, hormones (plasma renin, leptin, insulin, aldosterone, and cortisol), minerals,	
Angelaki et al. 2021 [38]	NA	Greece	528	44	61	Echocardiogram	>115 g/m^2^ (men)	16.8%	clinical variables (sex, age, BMI class, BSA, hypertension, and height	
							>95 g/m^2^ (women)		26 chosen ECG-derived features	Random Forest
Lim et al., 2021 [39]	Military	Singapore	17,310	100	18	Echocardiogram	>115 g/m^2^ (men)	0.8%	clinical variables were: body weight, height, body fat percentage, and systolic blood pressure	Logistic Regression, GLMNet, Random Forests, Gradient Boosting Machines
									ECG variables included: QT interval, mean QRS duration and R wave in lead I	
Zhao et al., 2022 [40]	Hospital based	China	3120	42	65	Echocardiogram	>115 g/m^2^ (men)	56%	uncertain	CNN
							>95 g/m^2^ (women).		Lab: Hgb, PLT, lipids, creatinine, Na, K	
Sammani et al., 2022 [41]	Hospital based	The Netherlands	2456	55	61	Echocardiogram	>115 g/m^2^ (men)	0.8%	age, systolic blood pressure and body surface area	XGBoost
							>95 g/m^2^ (women).		20 ECG data: p, QRS and T wave axes, pr, QRS, QT and QTc durations, peak amplitudes of p, Q, R, S and T waves	
Kokubo et al., 2022 [42]	Hospital based	Japan	12,008	64	57	Echocardiogram	>101 g/m^2^ for men	16.5%	19 factors—clinical (age, sex, height and weight) and ECG features (heart rate, rhythm, pr interval, QT interval. QRS axis, p wave axis	ENN
							>85 g/m^2^ for women		as well as QRS voltages in leads V1, V2, V5 and V6	LR, RF
Naderi et al., 2023 [43]	UK data base	UK	37,534	48	64	MRI	>70 g/m^2^ (men)	1.5%	Clinical—blood pressure, diabetes mellitus, lipids, cigarette and alcohol consumption	
							>55 g/m^2^ (women)		23 ECG variables from leads I, II, V1-6	LR, SVM, RF
Liu et al., 2023 [44]	Military Hospital	Tawain	952	90		Echocardiogram	>115 g/m^2^ (men)	18%	24 features which consisted of R peak and S valley amplitudes automatically obtained from the output of ECG signal	Decision tree SVM and Back propagated Neural Network

## Data Availability

The data used are available in the literature.

## Supplementary Materials

The following supporting information can be downloaded at: https://www.mdpi.com/article/10.3390/bioengineering11050489/s1, Figure S1: Preferred Reporting Items for Reviews and Meta-Analysis (PRISMA).

## Abbreviations

SVM	Support vector machine
LR	logistic regression
RF	Random Forest
ENN	Ensemble neural network
CNN	Convolutional neural network
DNN	Deep neural network
AUC	Area under the receiver operating curve
