# Supplementary figures and images for: Searching for the Best Machine Learning Algorithm for the Detection of Left Ventricular Hypertrophy from the ECG: A Review
Source: Bioengineering (Basel). 2024 May 15;11(5):489. doi: 10.3390/bioengineering11050489 (PMC11117908; doi:10.3390/bioengineering11050489)

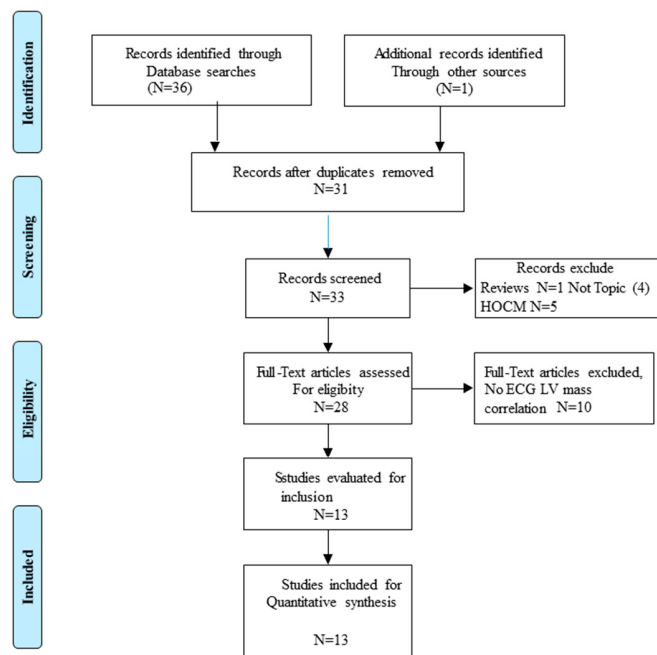

Figure S1: Preferred Reporting Items for Reviews and Meta-Analysis (PRISMA)

Supplement: Supplementary file 1 [file bioengineering-11-00489-s001.zip › bioengineering-2969742-supplementary.pdf]
